# Usefulness of Contrast Intracardiac Echocardiography in Performing Pulmonary Vein Balloon Occlusion during Cryo-ablation for Atrial Fibrillation

**DOI:** 10.1016/s0972-6292(16)30563-0

**Published:** 2012-12-02

**Authors:** Domenico Catanzariti, Massimiliano Maines, Carlo Angheben, Maurizio Centonze, Claudio Cemin, Giuseppe Vergara

**Affiliations:** 1 Division of Cardiology, S Maria del Carmine Hospital, Rovereto (TN), Italy; 2 Department of Radiology, S Chiara Hospital, Trento, Italy

**Keywords:** atrial fibrillation, cryoballoon ablation, intracardiac echocardiography, pulmonary vein isolation

## Abstract

**Background:**

Cryoballoon ablation (CBA) has been proven to be very effective for pulmonary vein (PV) isolation (PVI) if complete occlusion is achieved and conventionally assessed by angiographic injection of contrast within PV lumen. The aim of our study was to assess the usefulness of saline contrast intracardiac echocardiography in guiding CBA with respect to PV angiography.

**Methods:**

Thirty consecutive patients with paroxysmal atrial fibrillation were randomly assigned fluoroscopy plus color-flow Doppler (n = 15; group 1: an iodinated medium as both angiographic and echographic contrast) or contrast intracardiac echocardiography plus color-flow Doppler (n = 15; group 2: saline contrast) for guidance of CBA.

**Results:**

We evaluated 338 occlusions of 107 PVs. The intracardiac echocontrastography-guided assessment of occlusion, defined as loss of echocontrastographic back-flow to the left atrium after saline injection regardless of the visualization of PV antrum, showed a high level of agreement with the angiographic diagnosis of occlusion. PVI rate was similar in both groups and effectively guided by intracardiac echocontrastography (PVI using ≤ 2 double cryofreezes: 89% of PVs in group 1 vs. 91% in group 2; p=n.s.). Group 2 patients had significantly shorter procedure (127 ± 16 vs. 152 ± 19 minutes; p<0.05) and fluoroscopy times (30 ± 12 vs. 43 ± 9 minutes, p<0.05) and used a lower iodinated contrast (88 ± 26 vs. 190 ± 47 mL, p<0.05).

**Conclusions:**

PV occlusion and PVI during cryoablation can be effectively predicted by intracardiac saline echocontrastography. This technique reduces procedural time, radiological exposure and iodinated contrast use.

## Introduction

Pulmonary veins (PVs) electrical isolation (PVI) is the cornerstone of the ablative treatment of atrial fibrillation (AF) [1]. Radiofrequency energy sequential spot ablation is technically demanding and requires operator expertise and dexterity. The discontinuity of encircling lesions is a major cause of AF recurrences [2]. Cryothermal energy balloon ablation (CBA) has recently been introduced to overcome these limitations and can result in an equally effective and promising approach[3,4] for PVI as long as the complete occlusion of the PV antrum is pursued and confirmed by PV selective angiography [4-8].

Nevertheless angiography-guided CBA of PVs usually requires exposure to radiation doses and use of a contrast medium with the potential risks of inducing cancerous processes, allergic reactions and contrast nephropathy [9].

To reduce these risks, transesophageal echocardiography has been proposed [10], although general anesthesia is required in order to avoid patient discomfort.

More recently, intracardiac echocardiography [11-13] has been introduced to support CBA. A randomized comparison of transesophageal echocardiography versus intracardiac echocardiography has shown that the latter can increase the efficiency of CBA even during pull-down (PD) maneuvers [12].

A bolus of saline solution can be injected through the internal lumen of cryoballoon, thereby inducing the rapid appearance and diffusion of microbubbles within the left atrium. When the cryoballoon is positioned at the PV ostium, the absence of this contrastographic effect suggests that occlusion has been achieved.

The aim of our study was to prospectively assess the usefulness of saline contrast intracardiac echocontrastography in comparison with angiography (actual gold standard) in diagnosing PVs occlusion and in guiding CBA procedure.

## Methods

### Patients

Thirty consecutive patients with symptomatic drug-refractory paroxysmal AF, undergoing CBA, were enrolled in this study from October 2009 to December 2010. They provided written informed consent. Oral anticoagulation was administered for at least 4 weeks prior to the procedure, interrupted and replaced with low molecular weight heparin 5 days before hospital admission. A transesophageal echocardiography was performed within 24 hours of the procedure in order to exclude left atrial thrombus. Contrast-enhanced cardiac magnetic resonance imaging (MRI) [14] was performed prior to the procedure in order to generate a three-dimensional MRI reconstruction of the left atrium and PVs. Oral anticoagulation was resumed on the same day as the procedure. Exclusion criteria were: left atrial thrombus, left atrium echocardiographic diameter > 55 mm, heart failure and severe renal insufficiency (creatinine > 2.5 mg/dl). The study was approved by the review committee of the authors' institution. Patients were divided into 2 groups described in detail below.

### Cryoballoon ablation

The procedure was performed under conscious sedation and analgesia with fentanyl and midazolam. An 8/10-F ultrasound imaging catheter (AcuNav AcusonTM, Siemens AG) was inserted through a 9/11-F sheath, positioned in the right atrium and connected to an ultrasound platform (Acuson Sequoia C512, Siemens AG).

Using intracardiac echo-guidance, two trans-septal punctures [8] were performed. Both sheaths, were advanced to the left atrium and throughout the procedure were continuously flushed with heparinized saline solution, with the target being an activated clotting time of 250 to 350 seconds.

PV angiography was performed in two oblique projections (30 RAO, 45 LAO) to facilitate the following positioning of guide-wire and ablation catheter. PV angiographic ostium was defined as an abrupt angular change between the left atrium and the tubular portion of the PVs.

**Figure 1:**
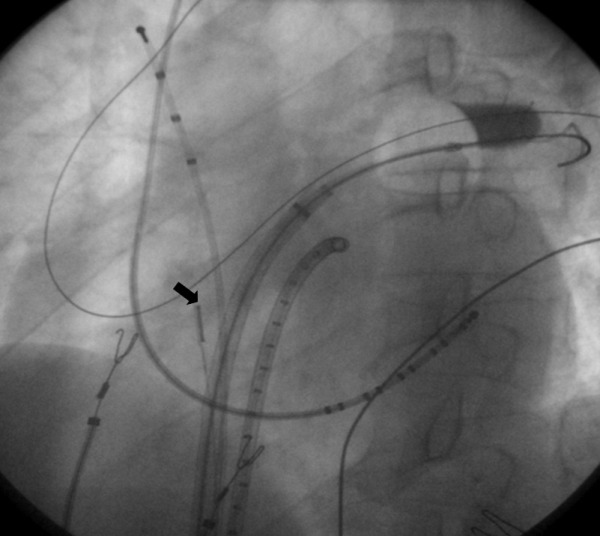
Angiographic occlusion of left upper pulmonary vein: contrast retention within the pulmonary vein ostium. Two transeptal sheaths are inserted within the left atrium. The black arrow shows the intracardiac echocardiographic catheter located in the right atrium with the optimal tilt and orientation necessary to visualize the left pulmonary veins.

A single 28-mm 'big-balloon' strategy was adopted in accordance with suggestions from Chung et al. [7]. Briefly, a stiff 0.035 mm guide-wire was positioned inside each PV (Amplatz Extra Stiff Wire Guide, Boston Scientific). A 28 mm double-walled cryoballoon (12 Fr Arctic Front, Medtronic) was advanced through a steerable sheath (15 Fr FlexCathTM, Medtronic Inc.) over the wire up to the left atrium, inflated and positioned at the antrum around the ostium of each PV, aiming at PVs occlusion (Figure 1). Each PV was frozen twice over 5 minutes in a wedge position. Further technical details are available in some previously published papers [5-8].

The internal lumen of the cryoballoon allows either iodinated contrast medium or saline solution to be injected in order to evaluate occlusion, using different imaging techniques according to the study protocol: fluoro-based and intracardiac echocardiography-based (echocontrastography and color-flow Doppler echocardiography). Multiple saline solution injections were used to cautiously advance the cryoballoon at the PV ostium to the most proximal wedged position, until the echocontrastographic effect disappeared and a brisk increase in resistance to manual injection was felt. Flushing of sheaths was temporarily stopped during saline injection and assessment of PV occlusion. Direct coaxial, hockey-stick and big-loop occlusive approaches were used, as already described [8].

In the case of failure of occlusion before freezing using the above-mentioned techniques, the PD maneuver was performed, aiming at occlusion during freezing [8,10,11,13]. PD consisted in the retiring of the balloon to achieve occlusion of the whole PV ostium after the early adhesion of the balloon surface to its superior border by freezing for 85 seconds.

### Assessment of Balloon Occlusion of PVs

Angiographic occlusion of PVs was defined as absence of contrast medium back-flow jet in the left atrium in multiple projections, contrast retention with dense delineation of the adjacent proximal portion of plugged PV and antegrade flow of contrast medium toward the PV tree periphery (Figure 1) [8].

Intracardiac echocontrastography occlusion of PVs was defined as the absence of the back-flow of microbubbles into the left atrium after rapid injection of 1-5 cc saline solution into the targeted PV within a timeframe of a few seconds.

**Figure 2:**
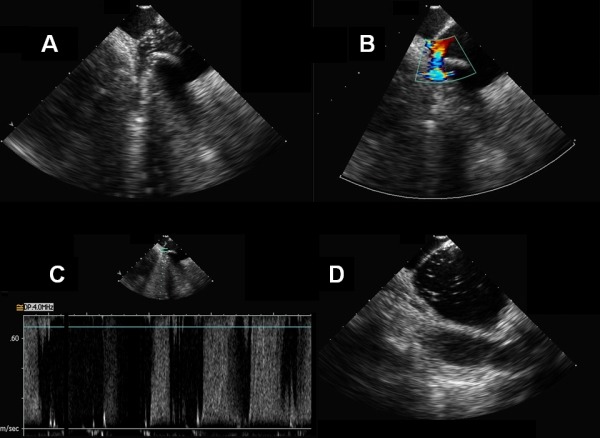
Contrast and Doppler intracardiac echocardiography: absence of occlusion. A. Contrast intracardiac echocardiographic view showing an inferior gap between right inferior pulmonary vein and the cryoballoon. By injecting saline solution through the balloon catheter central lumen, a back-flow of microbubbles is observed due to periballoon leak. B. Color flow Doppler visualization of turbulent leakage flow in the same pulmonary vein. C. Pulsed wave Doppler pattern of high-frequency monophasic leakage flow at the same anatomical gap. D. Same patient, left superior pulmonary vein: straightforward echocontrastographic effect of microbubbles back-flow into the left atrium after saline solution injection into the targeted pulmonary vein, despite the difficulty in analyzing the whole region of PV antrum by Doppler echocardiography.

Color-flow Doppler occlusion of PVs was defined if anatomic contact at the PV ostium-balloon interface was evident and no venous jets reflowing from residual gaps were observed [11-13]. In case of absence of PV occlusion, an attempt was made to locate the site of any leakage jet (Figure 2).

A high-velocity turbulent reflow jet was carefully distinguished from double-peaked accelerated compensatory flow originating from the adjacent PV by means of pulsed wave Doppler. PW Doppler peak flow velocities of all PVs were determined before and after ablation. Intra-ostial recordings from all PVs were made by a 14-pole circular catheter (Orbiter PV, Bard). After CBA, bidirectional electrical PVI was assessed in sinus rhythm and re-checked 30 minutes after the last application [15], using differential pacing when the left PV voltage amplitude was > 0.15 mV [16-18]. In the event of left atrium - PV residual electrical conduction, additional double cryoenergy applications were applied until persistent complete PVI could be documented. During the ablation of the right PVs, high-output phrenic nerve pacing was performed from the superior vena cava and, if diaphragm contraction disappeared, cryoenergy application was stopped in order to avoid phrenic nerve injury [6].

### Study groups

Study patients were randomly assigned to the following imaging support groups:

In Group 1 (n=15 patients) contrast medium injection was used to assess complete occlusion of targeted PV before freezing. Occlusion was assessed by angiography and intracardiac echocontrastography imaging. While the first electrophysiologist, evaluating the angiographic readings and handling the cryo-ablation catheter, was unable to see the echocontrastography readings, the second electrophysiologist, who was experienced in performing echocardiography, was unabled to see the angiographic readings. This was due to the necessary orientation and positioning of the ultrasound platform in respect to the fluoroscopy monitors in the operating room. CBA was carried out if angiography showed occlusion, irrespective of echocontrastography readings.

In Group 2 (n=15 patients) PV occlusion shown solely by real-time echocontrastography was required before freezing. However, in order to gather a larger number of data on the correlation between the imaging techniques, an angiographic bolus of contrast medium was also injected after echocontrastography confirmation of occlusion; this was done just at the onset of freezing, thereby not interfering in the decision-making process of cryoenergy delivery.

For PD-type cryofreezes, color flow-Doppler imaging alone was used to document the achievement of occlusion after freezing following the withdrawal of the cryoballoon, and to guide CBA in both groups, due to inadequacy of angiography and intracardiac echocontrastography readings.

### Study endpoints

Diagnostic agreement between angiography, which is regarded as the gold standard, and intracardiac echocontrastography in documenting occlusion before freezing was assessed in the overall population, in group 1 by means of single contrast medium injections and in group 2 by means of separate injections of saline solution and contrast medium. The efficiency of angiographic and echographic diagnoses of occlusion in guiding CBA was compared in the two groups by evaluating the achievement of PVI with ≤ 2 double cryofreezes for each PV. Finally, procedural time, radiological exposure and use of contrast medium were assessed in the 2 groups.

### Follow-Up

Follow-up visits performed at 2, 4, 6, 9, 12, 15, 18 and 21 months after ablation included clinical examination, periodic interviews, 12-lead surface ECG and 24-h ECG Holter monitoring. All AF episodes lasting >30 seconds that were documented after a 2-month blanking period were regarded as recurrences.

### Statistical Analysis

Mean ± standard deviation was used to describe continuous variables with normal distribution. Student's t-test for paired samples was applied for comparison. Categorical data were shown as absolute and relative frequencies and they were compared by means of Chi-squared or Fisher's exact test, as appropriate. The diagnostic agreement between angiography and intracardiac echocontrastography on the overall population, and in the A and B subgroups has been measured using the Kappa statistics. A p value <0.05 was considered statistically significant. All the analyses were performed using the statistical software SPSS 12.0 (SPSSInc, Chicago, Illinois).

## Results

### Patient Characteristics

Thirty-four patients were evaluated for inclusion in the study. Four patients were excluded from CBA owing to left atrium appendage thrombus (1), long-standing persistent AF (1), previous surgical repair of an atrial septal defect (1) or decompensated heart failure (1). The study therefore included 30 patients with symptomatic paroxysmal AF episodes. Patients' characteristics are detailed in Table 1.

Preprocedural MRI revealed a left common trunk in 13 patients (6 in group 1) and an accessory right pulmonary vein in 1 patient (group 1). In the 30 patients, all 108 PVs were analyzed by means of intracardiac echocardiography. In 78 PVs, the PV antrum was adequately explored by means of color flow-Doppler analysis; in the remaining 30 PVs, exploration was deemed to be insufficient. Ostial diameters of all PVs, as assessed by pre-procedural MRI, were not statistically different from those assessed by intracardiac echocardiography, as illustrated in Table 2. 

### Cryoballoon ablation

In both groups, a total of 107 out of 108 PVs were actually treated; one right inferior PV was not treated due to tachycardia-induced AF coming from the left upper PV (Table 3). In the 107 PVs treated, a total of 338 occlusions were achieved in the 2 groups including 74 occlusions regarding 13 common left sided ostia. Diagnostic agreement between echocontrastography and angiography with regard to the diagnosis of the presence/absence of occlusion was very high in the overall population and not statistically different in the two groups of patients (Global K Statistic = 0.98 p<0.001; group 1 K Statistic= 0.98 p<0.001; group 2 K Statistic= 0.95 p<0.001). There was no difference in the use of saline solution or iodinated contrast medium in the assessment of PV occlusion by intracardiac echocardiography.

Indeed, 285 occlusions (84%) were achieved before freezing; the remaining 53 occurred during PD-type maneuvers, in which occlusion was achieved after freezing (Table 3). Out of the 285 occlusions diagnosed by angiography, 283 (99%) were confirmed by echocontrastography. Only in 2 cases of occlusion (one per group) echocontrastography contradicted the diagnosis, in that it revealed a minimal echocontrastographic reflux in the left atrium, which was confirmed on color flow-Doppler by the reflux color-jet.

In 206 out of 283 cases (73%) occlusion was adequately evaluated also by color flow-Doppler and was confirmed in all cases; in the remaining cases (27%) color flow-doppler visualization was deemed insufficient or inadequate for the diagnosis of complete occlusion.

In 252 cases, angiography injections did not show occlusion before the beginning of freezing and were not followed by cryoenergy delivery. In 250 out of the 252 cases (99%), echocontrastography confirmed the angiographic absence of occlusion (clear contrastographic effect in the left atrium).

In all 107 of the PVs treated, PVI was achieved by means of CBA without the need for additional focal touch-up applications. Angiography in non-PD cryofreezes in group 1 and echocontrastography in non-PD cryofreezes in group 2 (both plus color flow-Doppler in PD-cryofreezes) proved equally efficient in guiding CBA, enabling PVI to be achieved by means of 1-2 double cryoenergy deliveries in 89% (48 of 54) and 92% (49 of 53) of PVs, respectively (Table 3).

In the remaining cases, another 3-4 applications of cryoenergy enabled PVI to be achieved in all the treated PVs.

In group 1 in which the diagnosis was guided by angiography, procedural time and radiological exposure time were longer and more iodinated contrast medium was used than in group 2 in which the diagnosis of pre-ablation occlusion was guided by saline contrast echocardiography (Table 3).

### Pull-down occlusive approaches

In 53 cases without initial demonstration of occlusion, PV was subsequently occluded after the onset of freezing, during a PD maneuver (Table 3). Neither angiographic nor echocontrastographic evaluation were able to confirm completion of the occlusion during cryoenergy delivery due to the freezing of the internal injection lumen. Color flow-Doppler evaluation, which was feasible in 46 of the 53 cases, always showed the achievement of occlusion after withdrawal during cryoenergy delivery. In the remaining 7 cases, in which color flow-doppler visualization also proved inadequate, the further 2-6ºC fall in temperature from the plateau level reached, following cryoballoon withdrawal, was the only sign deemed indicative of occlusion achievement [8,18].

During 53 PD maneuvers, 40 color flow-Doppler reflow jets were documented; the maximum velocity of the turbulent flow was ≥ 1.2 m/sec. Post-occlusion flow peak velocity of adjacent PVs transiently increased by 0.1-0.2 m/s, its maximum value being < 1.1 m/sec.

### Clinical follow-up

After a 2-month blanking period and a mean follow-up of 14.5±3 months, 20 (66%) patients were free from AF recurrences without antiarrhythmic therapy, and 3 patients (10%) were free from AF with antiarrhythmic therapy. No differences were observed between the 2 groups of patients (73% vs. 80%; p=n.s.).

### Complications

In one group 1 patient, the creatinine level transiently rose from a basal value of 1.8 to a peak value of 2.8. Pericardial effusion requiring drainage occurred in one patient of group 1. No acute PV stenosis occurred.

## Discussion

PVI remains the cornerstone for successful percutaneous AF ablation [1]. CBA has recently been proved to effectively induce PVI[5-8,19,20], when occlusion of the PV ostium is angiographically documented [4-7], although radiological exposure and the use of a contrast medium are not negligible and can increase the morbidity of the procedure. Prior studies have utilized the trans-esophageal [10] and intracardiac echographic [4,11-13,21] techniques based on color flow-Doppler assessment in cryoablation, with a view to reducing the above-mentioned adverse effects.

The trans-esophageal echocardiography has been adopted as a cost saving technique for supporting CBA. However, it requires general anesthesia due to the discomfort that it causes. In a study by Siklody et al.[10], the disappearance of the color-jet adjacent to the occluded PV displayed a good positive predictive value in inducing occlusion (achieved in 90 % of PVs) and in determining the PVI (achieved in 98% of occluded PVs vs. 9% of non-occluded PVs). However, this technique was seen to yield moderate or low visualization of 46% of PVs, owing to the poor echographic window. This may partly explain the lower rate of success in the inferior PVs (81% vs. 98%), which also required 2-3 additional focal applications of radiofrequency.

The use of intracardiac echocardiography during CBA [13] in 22 patients has been proved equally efficient in documenting occlusion both before cryoenergy delivery and during PD maneuvers. Schmidt et al. [12] in 22 patients compared angiography and color flow-Doppler, carried out through intracardiac echocardiography, showing that, in the color flow-Doppler group, exposure to radiation was lower as was the use of iodinated contrast medium. The above authors, however, reported limited visualization in 20% of cases (27% in our series). Nevertheless, the PVI success rate (98% of PVs) was very high, probably as a result of the use of a single 28 mm balloon and multiple approaches to occlusion.

### Main results

The present study compared the usefulness of saline solution echocontrastography in guiding CBA. We have observed that saline intracardiac echocontrastography can effectively guide CBA with a high diagnostic agreement in respect to angiography in assessing PV occlusion regardless of venous anatomy and echographic visibility. Moreover, procedural time, fluoroscopy and contrast use were reduced, showing a high predictive value with regard to PVI and allowing a low number of cryofreezes in 90% of PVs.

The diagnostic capability of the echocontrastographic method in documenting pre-ablation occlusion proved to be very high in both groups of patients. Saline solution echocontrastography proved feasible even in cases of moderate or poor color flow-Doppler visualization.

Indeed, the echocontrastographic occlusion is an "all or nothing" type of finding, based on the absence of microbubbles into the left atrium immediately after saline injection. As such, it does not depend on the availability of adequate visualization of the PVs, though admittedly the technique is not practical after the freezing of the internal lumen of the cryoballoon, as occurs in PD-type applications (16% of all applications in both groups).

Nevertheless, in 2 of the 285 occlusions, a diagnostic disagreement was observed. Despite the echocardiography diagnosis of complete occlusion, an intermittent reflux jet of contrast medium was seen after angiographic injection; this was due to the greater injection pressure in the venous chamber proximal to the occlusion, which enabled the contrast medium to seep through the seal between the cryoballoon and the PV ostium in an intermittent manner. However, echocardiographic reassessment by means of both color flow-Doppler and echocontrastography with saline injection documented complete occlusion. Indeed, unlike with angiography, a high pressure injection is not necessary in order to obtain an adequate contrastographic effect with the saline solution, and the injection can be repeated, even at different pressures, without fear of increasing the use of contrast medium. Although we regarded these two cases as false positive diagnoses of occlusion on echocontrastography, they could also be regarded as angiographic false negatives.

PVI, when supported by contrastographic intracardiac echocardiography integrated with color flow-Doppler, was achieved in a very high proportion of group 2 patients. This success rate has been practically identical to that recorded in group 1 patients, in whom the procedure was supported by color flow-Doppler-integrated angiography (98%), despite greater left atrial dimensions assessed by MRI were being observed in group 2 patients. Moreover, as demonstrated by the achievement of PVI in the 13 cases of left common trunk and the 1 case of right accessory PV, the venous anatomy made no difference to the therapeutic efficacy of intracardiac echocontrastography-supported CBA. In group 2 patients, in whom echocontrastography alone was used to support CBA in pre-ablation occlusive applications, radiological exposure and the overall use of iodinated contrast medium were reduced in comparison to group 1 patients with fluoroscopy guidance.

### Clinical implications

Our results led to the conclusion that the combined contribution of echocontrastography and color flow-Doppler may be used, though the spheres of application of each remain distinct. The ability of both techniques to diagnose occlusion and to predict PVI may be optimized within an integrated algorithm of imaging diagnostics during CBA, in which intracardiac echocontrastography would be used as an essential guide to initially occlusive applications (> 80% of all applications) while color flow-Doppler would fundamentally be dedicated to PD maneuvers.

Finally, intracardiac echocontrastography could be particularly useful in patients with iodinated contrast allergy.

## Limitations

This paper reports a single-center experience on a limited number of patients. A larger multi-center experience could attenuate the influence of inter-operator variability. 

The feasibility of performing CBA with almost exclusive echocardiographic support should be evaluated prospectively on a larger case series over longer periods of follow-up. 

Pre-procedural contrast MRI, routinely performed in our patients to achieve a 3D anatomical reconstruction of the left atrium and PVs, is not so strictly necessary in balloon-based ablation procedures.

Higher injection pressures were required for iodinated contrast due to its viscosity, despite this no differences in the contrastographic effects were observed using either iodinated or saline contrast. Intracardiac echocontrastography involves loss of anatomical information about distal PV anatomy and side-branches, provided by the iodinated contrast/fluoroscopic technique. However, in our experience the echocontrastographic effect is able to mitigate the diagnostic yield of this information.

## Conclusions

Intracardiac echocontrastography is feasible in documenting complete occlusion of the PV before freezing begins and it can therefore support CBA procedure.

This technique showed a high diagnostic agreement with angiography, the actual gold standard in documenting PV occlusion, and is not limited by either the type of echographic visualization or the venous anatomy.

Moreover, intracardiac echocontrastography markedly reduced procedural time, radiological exposure and use of iodinated contrast medium during the procedure. The best diagnostic yield could be obtained through a diagnostic algorithm that integrates echocontrastography with color flow-Doppler imaging, the latter being preferentially dedicated to pull-down approaches.

## Figures and Tables

**Table 1:**
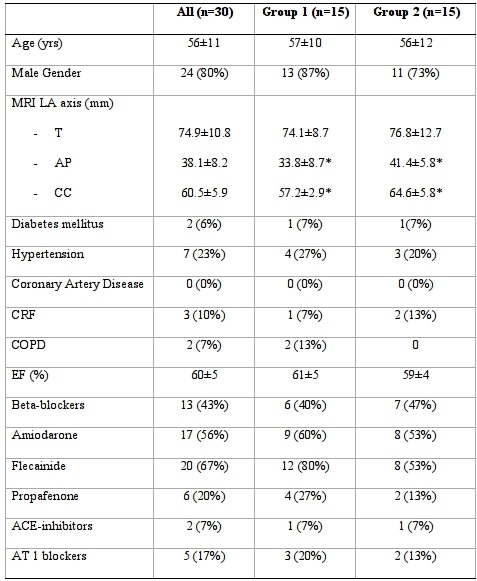
Patient Characteristics

*P<0.05 (group 1 vs group 2). MRI: magnetic resonance imaging; LA: left atrium; T: Transverse; AP: Antero-posterior; CC: cranio-caudal; CRF: chronic renal failure; COPD: chronic obstructive pulmonary disease; EF: ejection fraction. Values are mean ± standard deviation or n (%).

**Table 2:**
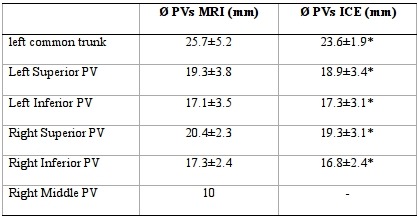
Maximal ostial diameters of PVs assessed by MRI and ICE

PV: pulmonary vein. ICE: intracardiac echocardiography . MRI: magnetic resonance imaging. Values are mean ± standard deviation. *P = n.s.

**Table 3:**
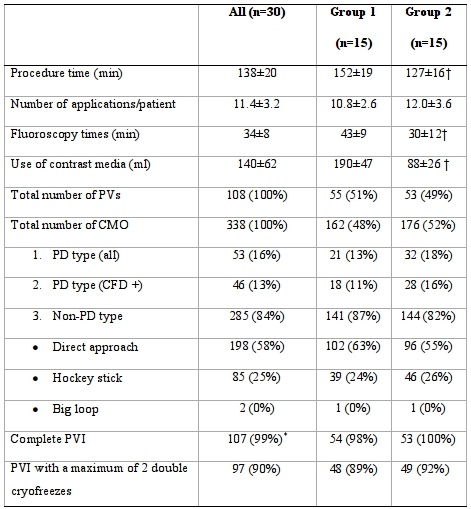
Procedural characteristics, occlusive approaches and electrophysiological endpoint

PVs: pulmonary veins; CMO: complete mechanical occlusion; PD: pull-down approach; CFD+ (in parentheses): CMO confirmed by Colour Flow Doppler, in which Colour Flow Doppler was considered adequate; PVI: pulmonary vein isolation. *1 Right inferior PV was not treated due to tachycardia-induced atrial fibrillation coming from left upper PV. Values are mean ± standard deviation or n (%). † P<0.05 (group 1 vs group 2; all others p = n.s.).
